# Exploiting morphobiometric and genomic variability of African indigenous camel populations-A review

**DOI:** 10.3389/fgene.2022.1021685

**Published:** 2022-12-12

**Authors:** Abdulmojeed Yakubu, Moses Okpeku, Ayoola J. Shoyombo, Gbolabo O. Onasanya, Lahouari Dahloum, Senol Çelik, Abolade Oladepo

**Affiliations:** ^1^ Department of Animal Science, Faculty of Agriculture, Centre for Sustainable Agriculture and Rural Development, Shabu-Lafia Campus, Nasarawa State University, Keffi, Nigeria; ^2^ Discipline of Genetics, School of Life Sciences, University of Kwa-Zulu Natal, Durban, South Africa; ^3^ Department of Animal Science, Landmark University, Omu-Aran, Nigeria; ^4^ Department of Animal Science, Federal University Dutse, Dutse, Nigeria; ^5^ Deparment of Animal Genetics and Breeding, Veterinary College and Research Institute, Tamil Nadu Veterinary and Animal Sciences University, Chennai, India; ^6^ Départment of Agronomy, Faculty of Natural Science and Life, Abdelhamid Ibn Badis, University, Mostaganem, Algeria; ^7^ Department of Animal Science, Faculty of Agriculture, Bingöl University, Bingöl, Turkey

**Keywords:** morphology, genetics, improvement, camels, Africa

## Abstract

Camels (*Camelus dromedarius*) in Africa are adapted to arid and the semi-arid environmental conditions, and are valuable for meat, milk and fiber production. On account of the growing demand for camels in this continent, there is a need for knowledge on their phenotypic and genetic diversity. This is fundamental to sustainable herd management and utilization including the design of appropriate breeding and conservation strategies. We reviewed studies on the phenotypic and genetic characterization, breeding objectives, systems of production, productive and reproductive performances, and pathways for the sustainable rearing and use of camels in Africa. The morphological and genetic diversity, productive and reproductive abilities of African camels suggest the existence of genetic variations that can be utilized for breeds/ecotypes’ genetic improvement and conservation. Possible areas of intervention include the establishment of open nucleus and community-based breeding schemes and utilization of modern reproductive technologies for the genetic improvement of milk and meat yields, sustainable management of rangelands, capacity building of the pastoralists and agro-pastoralists, institutional supports, formation of centralized conservation centres and efficient and effective marketing systems.

## Introduction

Camels (*Camelus dromedarius*), over the years, are renowned for quality milk, meat, and fibre production across the continents (Volpato and Howard, 2014; YEl Badawi, 2018; Gebremichael et al., 2019; Abri et al., 2019; Wilson, 2020a): They are tremendously important as sustainable species with specific attributes (milk composition, immune genes and health) (Burger, 2016; Abrhaley & Leta, 2018; Orazov et al., 2021; Anwar et al., 2022), draught ability, transportation means, and ecotourism thereby contributing to the economic empowerment, social-cultural wellbeing and food security of pastoralists and agro-pastoralists (Abdussamad et al., 2011; Kagunyu and Wanjohi, 2014; Traoré et al., 2014; Dioli, 2020; Wilson, 2020b; and c; Nagy et al., 2021). The animals are hardy and drought-resistant and have the ability to cope with certain climatic stresses and shocks (Watson et al., 2016; Robert, 2018). Camels also link continents through trades, and cultural activities (Burger, 2016). The 2020 FAO worldwide livestock data for camel populations were 38,654,378 heads out of which African countries contributed 33,691,906 heads (87.2%), respectively (FAOSTAT, 2022).

The unique traits camels exhibit in terms of morphology, adaptation, biochemistry, and behaviour are facilitated by natural and artificial selection (Alhaddad and Alhajeri, 2019; Berihulay et al., 2019). The camel genome harbors several unique variations (Holl et al., 2017; Ali et al., 2019; Tadesse et al., 2019), which are responsible for their survival in extreme climatic conditions (Ali et al., 2019). Since characterization of a breed is the initial step to its sustainable use; the extent of phenotypic and genetic variations is fundamental to the selection, improvement and utilization of various populations of camels in Africa (Bekele et al., 2018).

This review covers several features of the camels in Africa which include phenotypic traits, production systems, productive and reproductive attributes, genetic diversity and path ways to sustainable production, utilization and conservation of the animals.

## Domestication of camels

From the perspectives of ancient civilizations, the Old World camels (*Camelus dromedarius*) are important domestic animals which are found in arid and semi-arid Africa and the Middle East including western and central Asia (Marshall et al., 2014; Burger, 2016). The dromedary (one-humped camel) can also be found in the Sahelian States of West Africa, Sudan, Ethiopia, Somalia and northern Kenya (Teka, 1991; Nelson et al., 2015). They are different from the Bactrian camels (*Camelus bactrianus*) found mainly in the hot, cold steppes, and Central Asia deserts (Faye, 2020; Bornstein, 2021). The dromedary’s domestication occurred rather late in the history of human, probably before the Common Era (B.C.E.) (Uerpmann and Uerpmann, 2002).

**FIGURE 1:**
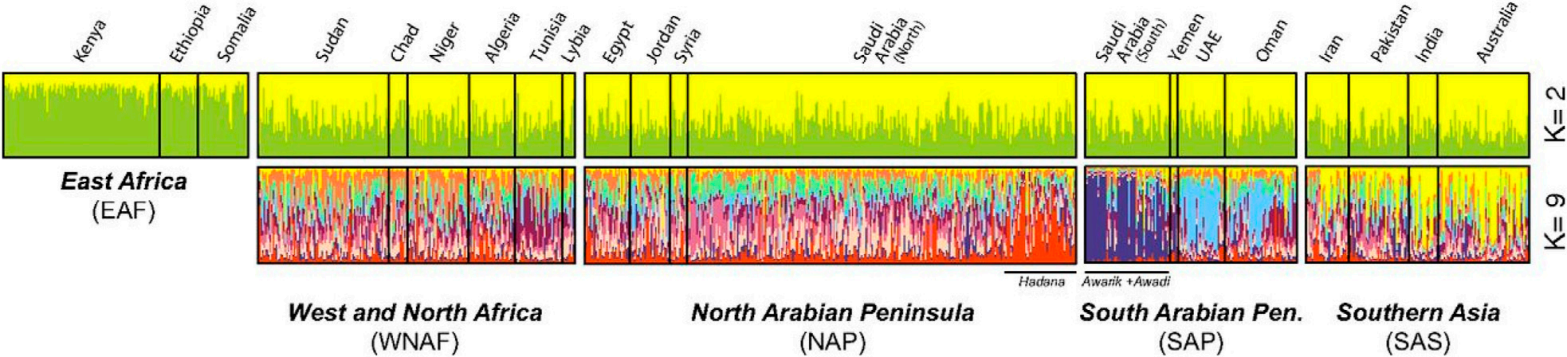
A structure analysis plot showing the domestication of African camels. Source: Almathen et al. (2016).

The genetics of the domestication of dromedary was reviewed by Orlando (2016), and was traced to the Arabian Peninsula, the Levant, north of Africa and beyond. This was supported by the microsatellites and mitochondrial markers of Almathen et al. (2016), coinciding with the rise of the Ottoman Empire some 600 ya. The findings of Almathen et al. (2016) microsatellite data also traced the routes of the modern dromedaries to two; the first is East Africa while the second encompasses all other regions (Figure 1). Ecogeographic barriers and cultural differences could have affected the domestication process of camels in East Africa. It is also possible that the domestication of camels could have been shaped by ‘‘positive selection of candidate genes underlying traits collectively referred to as ‘domestication syndrome’; and the core set of domestication genes is considerably smaller than the pan-domestication set’’ (Fitak et al., 2020). This implies that a lot of genetic variations exist in camels which could be exploited for the improvement of economically important traits.

## Phenotypic characterization of African camels

Phenotypic characterization of camels basically involves the qualitative and quantitative description of breed populations. The extent of phenotypic variation is important in the selection and utilization of different camel populations in breeding programs based on their specific characteristics and body conformation (Bekele et al., 2018).

## Qualitative traits

Based on the available records on qualitative physical traits, African camels can be characterized using coat colour, hair type, facial profile, nose shape, ear orientation and hump orientation (Table 1). The African camels can be distinguished based on twelve colour attributes namely, brown-black, dark-brown, sand-brown, grey-white, grey, whitish, creamish, red, yellow and pied. The hair is either smooth or rough while having straight, convex and concave facial profile. The ear is erect, horizontal or semi-pendulous. Flat nose shape was only reported in Borena camels in Ethiopia (Bekele et al., 2018) while hump of Nigerian indigenous camel was found to be erect (Tandoh et al., 2018). Udder size and teat size were either large, medium or rudimentary (Ishag et al., 2011). However, it is worthy of note that most populations of camels are referred to as breeds/ecotypes based on tribal affiliation/geographical location (Rosati et al., 2005; Meghelli et al., 2020).

**TABLE 1:** Physical qualitative traits of camels.

Traits	Attributes	Breeds/Ecotypes	Countries	Sources
Coat colour	Brown-black	Nigerian Indigenous	Nigeria	Abdussamad et al. (2015)
	Dark-brown	Nigerian Indigenous, Borena, Steppe, Sahraoui, Alsertaweya	Nigeria, Ethiopia, Algeria, Libya	Bakory, (2012); Abdussamad et al. (2015), Bekele et al. (2018), Meghelli et al. (2020)
	Sand-brown (Golden)	Nigerian Indigenous, Borena	Nigeria, Ethiopia	Abdussamad et al. (2015); Bekele et al. (2018)
	Brown	Steppe, Sahraoui, Maghrebi, Gabbra/Rendille, Sahilian, Lahawee	Algeria, Tunisia, Kenya, Eritrea, Sudan	Dioli, (2006); Tura et al. (2008); Ishag et al. (2011); Chniter et al. (2013); Meghelli et al. (2020)
	Grey-white	Nigerian Indigenous	Nigeria	Abdussamad et al. (2015)
	Grey	Turkana, Maghrebi, Nigerian Indigenous, Kenani	Kenya, Tunisia, Nigeria, Sudan	Tura et al. (2008); Ishag et al. (2011); Chniter et al. (2013); Tandoh et al. (2018)
	Whitish	Borena, Maghrebi, Annafi, Bishari	Ethiopia, Tunisia, Eritrea, Sudan	Dioli, (2006); Ishag et al. (2011); Chniter et al. (2013); Bekele et al. (2018)
	Creamish	Somali, Gabbra/Rendille	Kenya	Tura et al. (2008)
	Red	Steppe, Sahraoui, Hamra, Rashaidi	Algeria, Eritrea, Sudan	Mohamed, (2010); Dioli, (2006); Meghelli et al. (2020)
	Yellow	Maghrebi, Altebestee Almaharee	Tunisia, Libya	Bakory, (2012); Chniter et al. (2013)
	Fawn	Maghrebi	Tunisia	Chniter et al. (2013)
	Pied (Multicoloured)	Nigeria Indigenous, Geleb	Nigeria, Eritrea	Dioli, (2006); Abdussamad et al. (2015)
Hair type	Smooth	Borena, Maghrebi, Nigerian Indigenous	Tunisia, Ethiopia, Nigeria	Chniter et al. (2013); Bekele et al. (2018); Tandoh et al. (2018)
	Rough	Borena, Maghrebi, Nigerian Indigenous	Tunisia, Ethiopia, Nigeria	Chniter et al. (2013); Bekele et al. (2018); Tandoh et al. (2018)
Facial profile	Straight	Borena, Nigerian Indigenous, Kenani, Rashaidi	Ethiopia, Nigeria, Sudan	Ishag et al. (2011); Bekele et al. (2018); Tandoh et al. (2018)
	Convex	Borena, Nigerian Indigenous, Lahawee	Ethiopia, Nigeria, Sudan	Ishag et al. (2011); Bekele et al. (2018); Tandoh et al. (2018)
	Concave	Bishari	Sudan	Ishag et al. (2011)
Nose shape	Flat	Borena	Ethiopia	Bekele et al. (2018)
	Concave	Borena	Ethiopia	Bekele et al. (2018)
Ear orientation	Erect	Borena, Nigerian Indigenous	Ethiopia, Nigeria	Bekele et al. (2018); Tandoh et al. (2018)
	Horizontal	Borena, Alarabia	Ethiopia, Libya	Bakory, (2012); Bekele et al. (2018)
	Semi-pendulous	Borena	Ethiopia	Bekele et al. (2018)
Hump orientation	Erect	Nigerian Indigenous, Kenani, Rashaidi, Anafi, Bishari	Nigeria, Sudan	Ishag et al. (2011); Tandoh et al. (2018)
Udder size	Large	Rashaidi, Kenani, Lahawee	Sudan	Ishag et al. (2011)
	Medium	Kenani, Lahawee	Sudan	Ishag et al. (2011)
	Rudimentary	Anafi, Bishari	Sudan	Ishag et al. (2011)
Teat size	Large	Rashaidi, Kenani, Lahawee	Sudan	Ishag et al. (2011)
	Medium	Kenani, Lahawee	Sudan	Ishag et al. (2011)
	Rudimentary	Anafi, Bishari	Sudan	Ishag et al. (2011)

The reported diversity in qualitative physical traits of African camels might not be unconnected with territorial distribution and the historical and cultural context. Mammals’ pigmentation is determined primarily by eumelanin and pheomelanin distribution (Almathen et al., 2018). SNAI1 which interacts with MCIR, ASIP and KIT genes is known as a key player in the biological pathway of melanogenesis (Bitaraf Sani et al., 2022). It is generally believed that domestication is responsible for varying colour phenotypes (Volpato et al., 2017). The variation in coat colour in African camels could be a form of adaptive mechanism bearing in mind its suggested role in temperature regulation (Finch et al., 1984; Yakubu et al., 2010; Volpato et al., 2017). It has been reported that animals with dark colours absorbed more solar radiation than those with light colours; hence the former may perform lesser than the latter under hot environmental conditions (Fuller et al., 2016; Galván et al., 2018). Camels with white skin colour tend to have more platelets count in the blood than their black counterparts, with possible homeostatic responses that make them to thrive better in very hot environments (Al-Ghumlas, 2020). A contrary report, however, was submitted by Abdoun et al. (2013) where coat colour did not prove to influence heat tolerance in camels. This is an indication of a complex relationship between camel coat colour and the amount of radiant heat absorbed or reflected including the consequent heat load reaching the skin. This calls for more studies on the effects of coat colour and other coat characteristics on thermoregulation and heat tolerance in African camels. Camel hair has also been reported to play a role in photoprotection (Mauldin and Peters-Kennedy, 2016) and have temperature-regulating properties (Alibayev et al., 2020).

## Quantitative traits

Quantitative traits (body size and conformation) are useful in breed discrimination and establishment of phenotypic standards. It is globally important to assess the level of phenotypic variation in camel populations. This paves the way for proper documentation of the available gene pools that will permit the selection of elite animals for the production of superior individuals (Alhajeri et al., 2019; Ehsaninia et al., 2020; Atigui et al., 2021). Body weight (kg) and twenty linear body measurements (cm) of African camels varied among the different breeds/ecotypes, and between sexes. These ranged from 267.7 to 850.0 (male) and 248.4–717.6 (female) (body weight) to 51.7–70.2 (male) and 55.1–67.8 (female) (tail length) (Supplementary Table S1). The variations in the body parameters might be as result of the varying genetic make-up, adaptive capacities and management of the various breeds/ecotypes of camels. The dromedary populations are so different in size and zoometric traits that suppose genetic differentiation that is useful in terms of adaptation and performance (Harek et al., 2017). This provides a basis for selection of superior animals either for meat or milk production.

Camel bulls appeared to have higher values for size measures, which is an indication of sexual dimorphism (Tandoh and Gwaza, 2017). Such dimorphism could have resulted from the differential pressures (sexual- and natural-selection) experienced by both sexes (Yakubu et al., 2022). However, measurements done manually have some disadvantages such as stress of camels, less accuracy and livestock aggressiveness (leading to the attack of the individuals taking the measurements). In order to address these constraints, the use of geometric morphometrics to effectively capture the overall size and shape of camels has been proposed (Rahagiyanto et al., 2019; Gherissi et al., 2022). The many advantages of geometric morphometrics (three-dimensional) over the traditional method (one-dimensional) include proper account for size differences, provision of a more realistic image, easiness, less workload, and impersonal impartiality (Zelditch et al., 2012; Alhaddad and Alhajeri, 2019; Sénèque et al., 2019; Çağlı and Yılmaz, 2021; Gherissi et al., 2022).

## Breeding objectives and trait preferences

When setting a breeding objective, multiple traits are combined irrespective of whether one trait is dominant over others (Sölkner et al., 2008; Yakubu et al., 2019). All economically important traits should be considered in the breeding objective; thus, production and functional traits, the latter increasing profit by reducing costs, need to be taken into consideration. Farmers are also involved in setting up the breeding objectives (Ouédraogo et al., 2021; Wurzinger et al., 2021). In Gao, Mali, camels are mainly produced for milk (Traoré et al., 2014). Among Afar and Somali pastoral communities in Ethiopia, camels are important as a source of milk, meat, income, social status, culture and insurances (Tadesse et al., 2014; Gebremariam et al., 2020).

In different African countries, there are different ratings of criteria for the selection of animals for breeding purpose. The choice of camel breeding bull by pastoralists was mostly influenced by performance, conformation, and colour, while hump, temperament and size were less important in Nigeria-Niger Corridor (Abdussamad et al., 2011). In Oromia Regional State, Ethiopia, based on ranking (index), size/appearance (0.365), growth (0.354) colour (0.260) and libido (0.021) (Males) and Age at first calving (0.380), growth (0.287), size/appearance (0.255) and Colour (0.078) (Females) were the criteria used to select breeding animals (Bekele et al., 2018). Other traits of importance for selection of breeding camels include milk yield, coat colour, disease and parasite resistance, adaptation and bull that hails from more female-bearing ancestors (Mirkena et al., 2018; Gebremariam et al., 2020). According to Somalian herders, ‘adaptedness to harsh environmental conditions’ is of immense significance. The choice of this trait in Somaliland is mainly related to the relatively high droughts, the migration for pasture and water, and the changing climatic conditions (Marshall et al., 2016). Other highly ranked traits include the high market value of productivity attributes (meat and milk production and product quality (meat is of good quality/tasty). In Kenya, pastoralists preferred the Somali breed of Camels for increased milk yield and increased body size (Kuria et al., 2016). The most cited criteria for selection by herders in Mali were beauty, milk production, colour, disease resistance and speed (Traoré et al., 2014). In Algeria, Targui breed is reputed for its traction ability, while Sahraoui breed is more preferred for milk production (Mammeri et al., 2014). Similarly, agropastoralists in Burkina Faso are more disposed to camels with good traction ability (Andre et al., 2013). The Eritrean camels are highly ranked for their rough feed and water usage in arid environments (AU-IBAR, 2015).

## Production systems

The history of camel production systems has highly been on the migratory and extensive side. However, the systems have evolved over the years from a traditional system to a more diverse modern system of production. Camel’s husbandry in different parts of the world can be classified as traditional nomadic management system and transhumant, sedentary, semi-intensive and intensive management system (Kamili et al., 2020; Faraz et al., 2021). In Africa, camel livestock production systems can be broadly classified into two: ‘‘1) the traditional production systems (pastoral nomadic, pastoral transhumant, agro-pastoral and smallholder mixed crop-livestock) and 2) the modern production systems (ranching, intensive/semi-intensive peri-urban/urban, feedlot and commercial production)’’ (Mirkena et al., 2018). Predominantly, camels are kept in the pastoral and agro-pastoral production systems (Noor et al., 2013; Shishay and Mulugeta, 2018; Babege et al., 2021; Khalil et al., 2021; Machan et al., 2022). Basically, in the traditional pastoral systems, camels are housed in paddocks within the vicinities of the pastoralists in the night, and are allowed during the day to graze in communal land. The movement from one place to another is strategic, to enable the animals to have access to quality pastures, and to avoid conflicts with farmers (Traoré et al., 2014). Agro-pastoralism involves a combination of livestock rearing and crop production to a limited extent (Ishag and Ahmed, 2011; Ghude et al., 2017; Babege et al., 2021).

## Productive performance: Camel milk and meat production

Camel milk trait is very important in breeding considering the fact that on average, the milk produced by camels is six times more than that of the indigenous cattle in drylands (Oselu et al., 2022). Camel milk has some health benefits attributable to the consumption of quality desert plants (Faye, 2016). Camel meat is also highly valued for his nutritive and healthy contents (Kadim et al., 2014). In terms of milk production (hectogram per animal), camels from Ethiopia (8,900 hg/An), Kenya (6,800 hg/An), Mali (6,487 hg/An) and Niger (hg/An) appeared to have an edge over their counterparts from other African countries. However, meat yield was higher in camels found in Niger (3,002 hg/An), Kenya (2995 hg/An), Libya (2,948 hg/An) and Sudan (2,707 hg/An) (Table 2). The estimates obtained are based on the available 2020 records of FAOSTAT (2022). Meat production was based on yield/carcass weight while milk production was on yield of whole fresh milk. The variations in the milk and meat yields might be as a result of the varying genetic potentials, adaptability and management of the various camels breeds/ecotypes found in the reported African countries. These variations might be exploited in the genetic improvement of camels using superior animals from within or between countries. However, the importation of animals from one country to another should be done under the best global practices. This is to mainly avoid the transmission of transboundary diseases which might be counter-productive (Napp et al., 2018).

**TABLE 2:** Estimates of milk and meat production potentials of camels in Africa.

	Products (hg/An)	
Country	Milk	Meat
Algeria	1,551	1,472
Burkina Faso	1,080	1,914
Chad	1,311	1,800
Djibouti	1,980	1,501
Egypt	N/A	1,934
Eritrea	1,608	2,085
Ethiopia	8,900	1,796
Kenya	6,800	2,995
Libya	2,109	2,948
Mali	6,487	1,883
Mauritania	2,454	1,900
Morocco	2,708	2,154
Niger	4,635	3,002
Senegal	3,395	1,875
Somalia	3,987	1,700
Sudan	N/A	2,707
Tunisia	3,022	1,200

hg/An = Hectogram per animal**;** N/A = not available.

Source: FOASTAT (2022).

## Reproductive performance

Camel breeds seasonally (Khanvilkar et al., 2009). Reproductive information is essential in camel rearing for improved productivity. Available literature indicates differences in the reproductive performances of African camels under different environmental conditions especially in herds owed by nomadic and transhumant pastoralists:

In terms of breeding, male to female ratio ranged from 1:48–50 (Nigeria), 1:30–50 (Ethiopia), 1:40 (Algeria) to 1:50 (Kenya). Heifer and Bull mean ages at first mating were 3–3.9 and 5.63 years (Nigeria), 3.9–4.7 and 5.5–6.5 years (Ethiopia), 2.96 and 3.6 years (Algeria), 4-5 and 5-6 years (Kenya) and in males only, 5.64–7.4 years (Mali). Mean gestation period of 12.1–13 months (Nigeria), 12.8 months (Algeria), 12.5 months (Libya) has been documented. Age at first calving was 4.8–5 years (Nigeria), 5-6 years (Ethiopia), 4.3 years (Algeria), 5.2–6.4 years (Mali), 5-6 years (Kenya), 4.9–5.3 years (Niger), 7 years (Somalia), 6.5 years (Sudan), 5.5 years (Egypt) and 5.04 years (Mauritania) was reported. Calving interval was 23.8 months (Nigeria), 18–31.2 months (Ethiopia), 34 months (Somalia), 22.32 months (Algeria), 27-28 months (Kenya), 23–28 months (Sudan), 20.8–22.3 months (Egypt) and 12–48 months (Mauritania). The number of calvings in a lifetime was 8.49 calves (Nigeria), 8–11.6 calves (Ethiopia) and 7.8 calves (Egypt). The average birth weight for both female and male calves was 30 kg and 35 kg, respectively (Egypt). The mean herd’s annual fertility was 60% (Nigeria), 56.2% (Algeria) and 44.5% (Sudan), respectively (Wilson, 1989; Baumann and Zessin, 1992; Maillard, 1992; Bhakat and Sahani, 2000; Tezera et al., 2002; Ahmed Shek et al., 2005; Kaufmann, 2005; Al-Dahash and Sassi, 2009; Abdussamad et al., 2011; Traoré et al., 2014; Bakheit et al., 2016; Jaji et al., 2017; Mirkena et al., 2018; Gherissi et al., 2020; Gitao et al., 2021; Yassien et al., 2021; Ould Ahmed et al., 2022). However, the varying reproductive values reported in African camels could be due to factors such as genetic differences, agroecology, management and socio-cultural practices. Higher genetic merits of a breed/ecotype confers a superior advantage in terms of reproductive performance. Also, better rearing environment, especially where feed and water are available in the required amounts including reduction in the risks associated with the production systems will enhance reproductive performance (Kaufmann, 2005). However, the deliberate postponement of mating by the herder to extend the lactation length may affect herd population, while reduction in the age at first mating and the calving interval, including the control of reproductive disorders will increase the number of camels in the herd (Ali et al., 2018).

## Genetic and genomic characterization

Genetic diversity and population structure of camels will assist in sustainable management, selection and improvement of the herd (Legesse et al., 2018). Compared to other livestock species, data on genetic characterization of camels using molecular markers are limited (Nouairia et al., 2015). Some of the markers that are employed in assessing the genetic diversity and population structure of camels in Africa include:

## Microsatellites

The genetic diversity among the Ourdhaoui Médenine, Ourdhaoui Tataouine and Merzougui camel sub-populations in Tunisia using seven microsatellite markers revealed a mean number of alleles (MNA) of 6.5. The expected heterozygosity (H_e_) ranged from 0.76 to 0.84 while observed heterozygosity (H_o_) was 1.0. The sub-populations showed moderate genetic structure based on fixation index (FST = 0.052) (Nouairia et al., 2015). In four dromedary populations, Guerzni, Harcha, Khouari and Marmouri (Morocco), using 16 microsatellite markers; all loci were polymorphic, while the MNA was 13.4. The H_e_ ranged from 0.702 to 0.748, while the highest value of H_o_ was 0.699. The lowest genetic distance showed that the camels were well diversified (Piro et al., 2020).

Using 18 microsatellite loci in Nigerian indigenous camels, Abdussamad et al. (2015) found MNA of 5.61 per locus. The H_o_ and H_e_ were 0.52 and 0.63, respectively, indicating the level of genetic diversity. This could have resulted from continuous gene flow with other camel populations occasioned by transhumance along the Nigeria-Niger corridor. However, the molecular genetic data (AMOVA) did not clearly separate the populations into breeds based on coat colour. Tadesse et al. (2019) reported that MNA in six camel populations in Ethiopia ranged from 4.80 to 8.00 with an average of 6.80, while the H_o_ per population ranged from 0.44 (Gelleb) to 0.66 (Jigjiga) and the H_e_ ranged from 0.70 (Jigjiga) to 0.77 (Gelleb). Similar to the Nigerian camels, genetic analysis of microsatellite data did not show distinct genetic lineages based on geographical locations or designated camel breeds in Ethiopia (Legesse et al., 2018). Also, little differentiation existed between camels from southern Africa and Sudan. AMOVA showed that -0.09% of total variation resided between species, 0.26% between the two southern African populations and 99.83% within populations (Nolte et al., 2005). Cherifi et al. (2017) characterized Algerian and Egyptian (North African) camel populations using 20 Short Tandem Repeat (STR) markers. The nineteen polymorphic loci found displayed average MNA of 9.79. Average H_o_ was 0.611 while average H_e_ was 0.647. Gene diversity averaged over loci was 0.647. Meanwhile, the genetic differentiation of dromedary populations and geographic isolates in the two non-contiguous countries was weak. Overall, the inability to have a clear distinction into breeds using microsatellite loci could be as a result of genetic erosion which is a consequence of the historical use of camels as a cross-continental beast of burden along trans-Saharan caravan routes, as well as the traditional practices of the herders. These findings are congruous with the report of Almathen et al. (2016).

Contrastingly, Somali, Turkana, Rendille and Gabbra camel breeds of Kenya, and camel populations from Pakistan, Saudi Arabia and the United Arab Emirates, using fourteen microsatellite loci, were found to be genetically distinct. The H_e_ and allelic diversity values indicated that the Kenyan dromedaries were less diverse compared to their non-Kenyan counterparts (Mburu et al., 2003). In a related study, microsatellites revealed a defined global structure, separating East African and South Arabian camel populations from those of North Africa, North Arabia, and South Asia, respectively (Piro, 2021).

## Mitochondrial DNA

Mitochondrial DNA (mtDNA) depicts maternal inheritance pattern which can be used to show genetic variation between species (Piro et al., 2020; Alaqeely et al., 2021). The evaluation of variation of the mtDNA cytochrome b (cyto b) gene in Nigerian dromedary camels with other populations defined 37 haplotypes, including nine novel sequences from Nigeria (H3, H5–H7, H9–H11 and H13–H14), seven from Ethiopia (H26–H32), two each from Kenya (H35–H36) and Saudi Arabia (H1, H37), one from Turkey (H25), and 10 from Iran (H15–H24); while the remaining six were shared (Xueqi et al., 2021). Haplotypes, HT2 and HT12 were frequently shared by Nigerian camels and most other countries (Xueqi et al., 2021). However, in the 862bp-long mtDNAfragment of Nigerian camels, Abdussamad et al. (2015) detected 14 polymorphisms (13 transitions, one transversion) segregating into twelve haplotypes, and a haplotype (gene) diversity of 0.751. Legesse et al. (2018) reported that genetic analysis of cytochrome-b did not support any unique genetic lineage or distinct camel population structure in Ethiopia. However, Sulieman et al. (2014) revealed differences between the Kababeish, Shanabla and Nyalawei camel breeds of Sudan using mitochondrial DNA enzymes.

## Single nucleotide polymorphisms (SNPs)

In the recent times, the study of genetic diversity and polymorphisms of genes of economic importance involving the use of bio-makers have evolved with the use of advanced molecular techniques such as single nucleotide polymorphism (SNP). SNPs have been found as proven bio-markers for population-based screening of wide range of QTL in livestock animals (Onasanya et al., 2020, 2021; Galal-Khallaf et al., 2021). SNP genotyping of animals can be done using biochips. These chips vary from lower density to higher density (Klímová et al., 2020). Linkage disequilibrium (LD) between markers and QTL can better be captured using denser chips or panels (Aliloo et al., 2018). SNP richness is the number of SNPs with typical or common frequencies (Ma et al., 2020).

In a study to estimate the polymorphism of the tyrosinase (TYR) gene among Maghrebi, Sudani, Somali, and Falahy camel breeds of Egypt, it was observed that The CT heterozygote genotype had the highest frequency of 45%. The C/T SNP of TYR gene was associated with neck length, height at withers and chest girth, teat separation and teat floor distance traits (Nowier et al., 2020). Abd El-Aziem et al. (2015) detected a SNP (C264T) in growth hormone (GH) gene with possible association with higher growth rate in Fallahi, Maghrabi, Mowalled, Sudany and Somali breeds of camels in Egypt. Ishag et al. (2010) reported SNP 419C>T in the non-coding region (intron 1) of GH gene of Kenani, Lahwee, Rashaidi, Anafi, Bishari and Kabbashi camel breeds of Sudan. This could aid in the determination of genetic relationships among the different camel breeds. Similar SNP (C/T) was found at positions 062 and 232, respectively in Nigerian indigenous camels (Ibrahim et al., 2022). In a related study of the myostatin (MSTN) gene, which affects muscularity (Ramadan and Inoue-Murayama; Bruno et al., 2022) using twenty two camel samples from Tunisia, Algeria, and Egypt, Muzzachi et al. (2015) found at intron 1, G486C, G798A and C799T substitutions. This could be a reflection of the peculiarity of the evolutionary history of camels in the three north African countries. Also in Egypt, EL-Kholy et al. (2016) reported in camels that a SNP (A377T) of myogenic factor 5 (MYF5) gene having a frequency of 0.67, was associated with carcass width at brisket and fat thickness of longissimus dorsi.

Genetic diversity of three variants of casein gene in five camel ecotypes of Tunisia revealed SNPs, c.150G>T at CSN1S1 (αS1-casein), g.2126A>G at CSN2 (β-casein), and g.1029T>C at CSN3 (κ-casein) (Letaief et al., 2022). When the results obtained were compared with published data on camels from Sudan and Nigeria, H_o_ and local inbreeding differences were observed across the populations from Tunisia, Sudan, and Nigeria. The H_o_ ranged from 0.194 to 0.327 (Tunisian), 0.293–0.406 (Sudanese) and 0.357 (Nigerian) (Letaief et al., 2022). However, in United Arab Emirates (UAE), an Asian country, using targeted next-generation sequencing, most variants of the casein gene were located in the non-coding introns and upstream sequences, but a few variants showed the possibility of functional impact. CSN2 was found to be most polymorphic, with a total of 91 different variants, followed by CSN1S1, CSN3 and CSN1S2, respectively. CSN1S1, CSN1S2 and CSN2 each had at least two variants, while one functional allele was found in CSN3 (Mutery et al., 2021). Future research efforts should target the functional and structural impact of these variants.

## Whole genome sequencing (WGS)

Mammals inhabiting deserts show remarkable adaptive traits that have evolved repeatedly and independently in different species (Ali et al., 2019; Rochas et al., 2021). There is dearth of information on WGS of African camel breeds/ecotypes. However, in a study involving the full genome sequence data of six camels from the Arabian Peninsula and the genotyping-by-sequencing data of forty four camels from Sudan, genome admixture and principal component analyses indicated distinct geographic separation between the camels from Sudan and those from the Arabian Peninsula (Bahbahani et al., 2019). However, there was no specific within-population genetic distinction. Higher mean heterozygosity (0.560) was obtained in Arabian Peninsula camels compared to the value of 0.347 of the Sudanese. Pooled heterozygosity revealed 176, 189, and 308 candidate regions under positive selection in the Sudanese camel populations. These regions (Table 3), according to Bahbahani et al. (2019), are believed to host genes that might be associated with adaptation to arid environment, dairy traits, energy homeostasis, and chondrogenesis.

**TABLE 3:** Candidate regions of genes associated with functional traits in camels.

Functional category	Gene ID	Gene description	Type of candidate region
Immune response	C9	Complement component C9	Packing/racing/all Sudan camels (Hp region)
	IL6R	Interleukin-6 receptor subunit alpha	Packing/racing/all Sudan camels (Hp region)
	CCR8	C–C chemokine receptor type 8	Packing/all Sudan camels (Hp region)
	CX3CR1	CX3C chemokine receptor 1	Packing/racing/all Sudan camels (Hp region)
	LOC105100014	Complement receptor type 1-like	Racing camels/all Sudan camels (Hp region)
	C1QTNF8	Complement C1q tumor necrosis factor-related protein 8	Packing/all Sudan camels (Hp region)
Fertility	LOC105094930	Olfactory receptor 1S1-like	Racing camels (Hp region)
	LOC105094932	Olfactory receptor 5B12	Racing camels (Hp region)
	LOC105094933	Olfactory receptor 5B3-like	Racing camels (Hp region)
	ESR1	Estrogen receptor	Racing camels (Hp region)
	SPACA5	Sperm acrosome-associated protein 5	Packing/racing/all Sudan camels (Hp region)
Milk content	PICALM	Phosphatidylinositol-binding clathrin assembly protein	Packing/racing/all Sudan camels (Hp region)
Chondrogenesis	LOC105087163	Chondroitin sulfate proteoglycan 4-like	Packing/racing/all Sudan camels (Hp region)
	CRLF1	Cytokine receptor-like factor 1	Fst region
	CHSY1	Chondroitin sulfate synthase 1	Racing (Hp region)
Energy homeostasis	ESRRG	Estrogen-related receptor gamma	Packing/racing/all Sudan camels (Hp region)
	CRTC1	CREB-regulated transcription coactivator 1	Fst region
Running performance	NAA16	N (alpha)-acetyl transferase 16	Fst region

Hp = pooled heterozygosity; Fst = genetic differentiation.

Source: Bahbahani et al. (2019).

In another study, Khalkhali-Evrigh et al. (2018) reported that 4,727,238 SNPs and 692,908 indels (insertions and deletions) were detected by aligning sequenced nucleotides to the dromedary consensus. In-silico functional annotation of the discovered SPN variants in the studied two Iranian female dromedary camel samples showed that most SNPs (2,305,738; 48.78%) and indels (339,756; 49.03%) were located in intergenic regions. A comparison of the detected SNPs with those of the African camel indicated that they had 993,474 SNPs in common. The authors further found 15,168 non-synonymous SNPs in the shared SNP variants of the three camels and hypothesized that detected SNPS could affect the function and structure of protein.

## Genetic parameters estimation

Estimates of heritability, repeatability and breeding values of meat yield, milk yield, growth measures, reproductive and adaptive traits are lacking in African camels, unlike in others (Bene et al., 2020). The only available information was obtained on 302 records on lactation over a 10-year period for a herd of camels in Egypt with respect to total milk yield (TMY), day milk yield (DMY) and length of lactation period (LP). Estimates of additive heritability (h^2^a) for TMY, DMY and LP were 0.25, 0.30, and 0.17, respectively. Medium repeatability value (0.19) was obtained for LP, while higher values (0.36 and 0.43) were recorded for TMY and DMY, respectively. The predicted breeding values of the camels for TMY, DMY, and LP were 143.07 kg, 1.5 kg and 113.9 days, respectively (Shehab El-Din et al., 2022). These are potential values for the genetic improvement of camels.

## Pathways towards sustainable camel production and conservation in Africa

### Genetic improvements

In order to utilize the potential of camels, there is a need for genetic improvement using marker assisted selection while sustaining their genetic diversity and resilience (Al Abri and Faye, 2019; Berghof et al., 2019; Burger et al., 2019). Genetic improvement of camels will lead to increased production and productivity including the development of breeds specialized for different purposes (meat, milk, and leather).

A proposed breeding scheme for camels under the traditional systems can be operated on a communal or cooperative basis, and could involve the implementation of an open nucleus breeding programme (Seliaman et al., 2013). The use of open nucleus system (Oseni et al., 2017) offers a simple procedure and is particularly advantageous because it will promote exchange of elite animals between communities (Oseni et al., 2017), thereby stemming the tide of inbreeding within villages, and facilitate conservation and diversity in the gene pool of camels.

It is also imperative to establish community-based breeding programs (CBBPs) (incorporating indigenous knowledge), involving all relevant stakeholders. The scheme is aimed at initiating systematic breeding at the community level, including an organized animal identification and recording of performance and pedigree data (Wurzinger et al., 2021). The breeding plan should involve incorporating the breeding objectives and traits of preference including the socio-cultural practices of the pastoralists. This is because vast majority of camels in Africa are traditionally managed. Similarly, Harek et al. (2022), reported that the biodiversity of camels can be safeguarded with the full support and integration of the herders in any breeding programs.

For industrial use of camels, especially where there are adequate resources, genetic improvement of local breeds/ecotypes utilizing modern reproductive technologies (Artificial insemination (AI) and embryo transfer (ET) can be exploited. These tools play an effective role in propagating improved breeds (Köhler-Rollefson, 2022).

### Development of rangelands and sustainable management practices

Rangeland is important for the agroecosystem. Rangelands help in the preservation of biodiversity and some of the species of plants that are used for medical purposes. Rangeland policies and implementations with respect to conservation are necessary for sustainable management (Coppock et al., 2022; Farid et al., 2022; Jamil et al., 2022; Machan et al., 2022). It will assist in the nutrition of camels for improvement in their productive and reproductive performances (Kuria et al., 2021). This becomes more imperative considering the shift in herd composition favouring camels in areas with expected environmental challenges: This is because milk production from cattle-based dairy systems in sub-Saharan Africa is increasingly under stress due to climate change, thereby threatening food security and livelihoods (Rahimi et al., 2022). According to Faye (2022), changes associated with climate including the need to globalize the economy of the world call for the intensification of camel production, especially in African countries such as Chad, Mali, Niger, Senegal, Nigeria, Burkina Faso, Cameroon, Tanzania, and Uganda. The benefits of the intensification of camel farming have been buttressed by Nagy et al. (2022).

### Capacity building on camel production and management

To improve livestock production and productivity, there is a need for the capacity building of the herders. Knowledge improvement in the management, health, breeding, proper decision making and problem solving will not only increase production, but the profitability of the camel enterprise ((Asiimwe et al., 2020; Kuria et al., 2021). These activities can be efficiently and effectively executed through cognate extension services on the parts of the governments and non-govermental organizations (Salamula et al., 2017). Asadzadeh et al. (2022) reported that the acquisition of knowledge on herds’ communication and networking, including camels’ phenotyping, and tracking resulted in good herd’s performance.

#### Institutional supports for camel production and conservation

Assisting herders in all possible ways will stimulate interests and facilitate increase in camel characterization and production. These include facilitating the conduct of appropriate high quality research targeting the needs of the herders, marketers and consumers; policy and development interventions; legal frameworks, infrastructural supports (good roads, access to potable water and electricity), and giving incentives to herders in terms of credit facilities and inputs. Such assistance can be made possible by national, regional and international relevant institutions including donor agencies and developmental organizations (Leroy et al., 2015; Bediye et al., 2018; Vanvanhossou et al., 2021).

#### Establishment and maintenance of camel populations in centralized breeding flocks

This involves both *in situ* (on-farm) and *ex situ* (institutional farms anivate breeding farms) conservation. This is to prevent camels from going into extinction. The on-farm (community-based conservation) program should involve the local herders. This is because such bottom-top approach will give the herders sense of belonging, which we make them readily accept and work towards the successful conservation of the camels. According to Ovaska et al. (2021), for conservation to succeed, there must be individuals who are both willing and able to keep livestock breeds. This process will permit monitoring which requires the regular checking of the status of breeds/populations, evaluation of their trends in the size and structure, their ecological distribution, production systems, and genetic diversity ((FAO, 2011). However, conserving breeds by establishing dedicated farms involves a substantial investment in infrastructure and other resources (FAO, 2013).

#### Development of effective and efficient marketing chain for camels

The essence of establishing any camel enterprise(s) is to produce for consumption and sales in order to contribute to food security and improve the livelihoods of the increasing rural, peri-urban and urban populations. Therefore, there is a dire need for strong and reliable marketing channels for the sales of live animals and animal products such as milk and meat. In this wise, there should be a synergy in the activities of herders, middlemen, cooperative groups and governments in ensuring that the best quality animals and their products get to the markets. Additionally, there should be price control mechanism to ensure that the herders and other stakeholders within the marketing chain do not run at a loss. This will go a long way in guaranteeing sustainable production of camels which could contribute to the gross domestic products (GDPs) of African countries. Similar submissions have been made on the need for herders, middlemen, the cooperatives and the governments to jointly promote camel meat and milk businesses (Gebremichael et al., 2019; Zakaria et al., 2020; Kena, 2022; Oselu et al., 2022).

## Conclusion

Camel populations in Africa have some phenotypic, productive and reproductive attributes including genetic variations, based on the polymorphic nature of the loci reported. However, future genetic studies using robust methods are required to unravel the admixture process between them for proper breeds/ecotypes’ differentiation. Also, there is a need for interventions such as appropriate genetic improvement programs, utilisation of modern reproductive technologies, rangeland conservation, capacity building on sustainable camel production, institutional supports, establishment of centralized conservation stations, and strong marketing strategies. Such interventions are essential for the preservation, utilization and genetic improvement of camels geared towards increased production and productivity including higher profit.

## Data Availability

The original contributions presented in the study are included in the article/Supplementary Material, further inquiries can be directed to the corresponding authors.
