# Supplementary material for: Exploiting morphobiometric and genomic variability of African indigenous camel populations-A review
Source: Front Genet. 2022 Dec 12;13:1021685. doi: 10.3389/fgene.2022.1021685 (PMC9791103; doi:10.3389/fgene.2022.1021685)
Supplement: Supplementary file 1 [file Table1.docx]

Supplementary Table 1. Body weight (kg) and biometric traits (cm) of adult camels

| Traits | Mean values | Breeds/Ecotypes | Countries | Sources |
| --- | --- | --- | --- | --- |
| Body weight | M: 267.7-850.0 | Kenani, Rashaidi, Lahwee, Anafi, Bishari, Kabbashi, Liben, Steppe, Sahraoui, Maghrebi, Somali, Nigerian Indigenous | Sudan, Ethiopia, Algeria, Tunisia, Kenya, Nigeria | Kamoun, 2005; Ishag et al., 2010, Yosef et al., 2014, Tandoh et al., 2018; Meghelli et al., 2020, Oselu et al., 2022. |
|  | F: 248.4-717.6 | Aftout, Kenani, Rashaidi, Lahwee, Anafi, Bishari, Kabbashi, Gelleb, Steppe, Sahraoui, Maghrebi, , Nigerian Indigenous | Mauritania, Sudan, Ethiopia, Algeria, Tunisia, Nigeria | Kamoun, 2005; Ishag et al., 2010; Ould Ahmed et al., 2022; Yosef et al., 2014; Tandoh et al., 2018; Meghelli et al., 2020 |
|  | Combined: 258.0-550.0, | Gabbra/Rendille, Turkana, Somali, Steppe, Sahraoui, Southern Algeria | Ethiopia, Kenya, Algeria | Grund, 2004; Tura et al., 2008; Boujenane, 2019; Meghelli et al., 2020 |
| Body length | M: 148.7-164.4 | Jigjiga, Issa, Hoor, Ayden, Liben, Borena, Kerreyu, Afar, Nigerian Indigenous | Ethiopia, Nigeria | Legesse et al., 2018; Tandoh et al., 2018 |
|  | F: 140.0-162.7 | Maghrebi, Aftout, Jigjiga, Issa, Hoor, Ayden, Liben, Borena, Kerreyu, Afar, Nigerian Indigenous | Tunisia, Mauritania, Ethiopia, Nigeria | Chniteret al., 2013; Legesse et al., 2018; Tandoh et al., 2018; Ould Ahmed et al., 2022 |
|  | Combined: 132.4-143.9 | Borena, Steppe, Sahraoui | Algeria, Ethiopia | Meghelli et al., 2020, Kebede et al., 2022 |
| Height at withers | M: 176.5-210.9 | Jigjiga, Issa, Hoor, Ayden, Liben, Borena, Kerreyu, Afar, Steppe, Sahraoui, Nigerian Indigenous | Ethiopia, Algeria, Nigeria | Legesse et al., 2018; Tandoh et al., 2018; Meghelli et al., 2020; |
|  | F: 170.0-210.9 | Rendille, Maghrebi, Jigjiga, Issa, Hoor, Ayden, Liben, Borena, Kerreyu, Afar, Aftout, Nigerian Indigenous | Tunisia, Kenya, Ethiopia, Mauritania, Nigeria | Grund, 2004; Chniteret al., 2013; Legesse et al., 2018; Tandoh et al., 2018; Gebremariam et al., 2020, Ould Ahmed et al., 2022 |
|  | Combined: 175.9-185.0 | Steppe, Sahraoui, Bishari, Arabi, Rashaidi, Anafi, Kenani | Algeria, Sudan | Osman et al., 2015; Meghelli et al., 2020 |
| Fore limb length | M: 148.4-157.3 | Jigjiga, Issa, Hoor, Ayden, Liben, Borena, Kerreyu, Afar, Nigerian Indigenous | Ethiopia, Nigeria | Legesse et al., 2018; Tandoh et al., 2018 |
|  | F: 148.3-154.3 | Jigjiga, Issa, Hoor, Ayden, Liben, Borena, Kerreyu, Afar, Nigerian Indigenous | Ethiopia, Nigeria | Tandoh et al., 2018; Legesse et al., 2018; Gebremariam et al., 2020, |
|  | Combined: 148.3 | Jigjiga, Issa, Hoor, Ayden, Liben, Borena, Kerreyu, Afar | Ethiopia | Legesse et al., 2018 |
| Hind limb length | M: 155.9-170.8 | Jigjiga, Issa, Hoor, Ayden, Liben, Borena, Kerreyu, Afar, Nigerian Indigenous | Ethiopia, Nigeria | Tandoh et al., 2018; Legesse et al., 2018 |
|  | F: 163.8-169.7 | Afar, Nigerian Indigenous | Ethiopia, Nigeria | Tandoh et al., 2018; Gebremariam et al., 2020 |
|  | Combined: 174.5-197.7 | Sahraoui, Targui, Jigjiga, Issa, Hoor, Ayden, Liben, Borena, Kerreyu, Afar | Algeria, Ethiopia | Belkhir et al., 2013; Legesse et al., 2018 |
| Foreleg hoof circumference | M: 95.6 | Shinille | Ethiopia | Yosef et al., 2014 |
|  | F: 72.8 | Hoor | Ethiopia | Yosef et al., 2014 |
|  | Combined: 59.4 | Borena | Ethiopia | Kebede et al., 2022 |
| Hind leg hoof circumference | M: 87.8 | Shinille | Ethiopia | Yosef et al., 2014 |
|  | F: 69.9 | Liben | Ethiopia | Yosef et al., 2014 |
|  | Combined: 52.2 | Borena | Ethiopia | Kebede et al., 2022 |
| Heart or Chest girth | M: 202 .0-207.2 | Nigerian Indigenous, Kenani, Rashaidi, Lahwee, Anafi, Bishari, Kabbashi | Nigeria, Sudan, | Ishag et al., 2010; Tandoh et al., 2018 |
|  | F: 173.4-236.1 | Afar, Aftout, Kenani, Rashaidi, Lahwee, Anafi, Bishari, Kabbashi, Nigerian Indigenous | Ethiopia, Mauritania, Sudan, Nigeria | Ishag et al., 2010; Gebremariam et al., 2020; Ould Ahmed et al., 2022; Tandoh et al., 2018 |
|  | Combined: 159.0-208.0 | Kenani, Rashaidi, Lahwee, Anafi, Bishari, Kabbashi, Steppe, Sahraoui, Borena | Sudan, Algeria, Ethiopia | Ishag et al., 2010, Meghelli et al., 2020; Kebede et al.2022 |
| Depth of chest | M: 75.6-82.0 | Jigjiga, Issa, Hoor, Ayden, Liben, Borena, Kerreyu, Afar, Hoor | Ethiopia | Yosef et al., 2014; Legesse et al., 2018 |
|  | F: 75.6-80.6 | Jigjiga, Issa, Hoor, Ayden, Liben, Borena, Kerreyu, Afar, Gelleb | Ethiopia | Yosef et al., 2014; Legesse et al., 2018 |
|  | Combined: 51.4-80.6 | Jigjiga, Issa, Hoor, Ayden, Liben, Borena, Kerreyu, Afar, Gelleb | Ethiopia | Yosef et al., 2014; Legesse et al., 2018; Kebede et al., 2022 |
| Width of chest | M: 51.4-63.5 | Jigjiga, Issa, Hoor, Ayden, Liben, Borena, Kerreyu, Afar, Gelleb | Ethiopia | Yosef et al., 2014; Legesse et al., 2018 |
|  | F: 51.1-63.6 | Jigjiga, Issa, Hoor, Ayden, Liben, Borena, Kerreyu, Afar, Gelleb | Ethiopia | Yosef et al., 2014; Legesse et al., 2018 |
|  | Combined: 37.1-63.5 | Jigjiga, Issa, Hoor, Ayden, Liben, Borena, Kerreyu, Afar, Gelleb | Ethiopia | Yosef et al., 2014; Legesse et al., 2018; Kebede et al., 2022 |
| Barrel girth | M: 170.0-265.3 | Jigjiga, Issa, Hoor, Ayden, Liben, Borena, Kerreyu, Afar, Kenani, Rashaidi, Lahwee, Anafi, Bishari, Kabbashi, Nigerian Indigenous | Ethiopia, Sudan, Nigeria | Ishag et al., 2010; Yosef et al., 2014; Legesse et al., 2018; Tandoh et al., 2018 |
|  | F: 178.0-263.3 | Jigjiga, Issa, Hoor, Ayden, Liben, Borena, Kerreyu, Afar, Gelleb, Kenani, Rashaidi, Lahwee, Anafi, Bishari, Kabbashi, Aftout, Nigerian Indigenous | Ethiopia, Sudan, Mauritania, Nigeria | Yosef et al., 2014; Legesse et al., 2018; Ishag et al., 2010; Tandoh et al., 2018; Ould Ahmed et al., 2022 |
|  | Combined: 163.8-263.7 | Bishari, Arabi, Rashaidi, Kabbashi, Lahwee, Anafi, Kenani, Jigjiga, Issa, Hoor, Ayden, Liben, Borena, Kerreyu, Afar, Sahraoui, Targui, | Sudan, Ethiopia, Algeria | Ishag et al., 2010; Belkhir et al., 2013; Yosef et al., 2014; Osman et al., 2015; Legesse et al., 2018; Kebede et al., 2022 |
| Neck length | M: 106.7-116.0 | Jigjiga, Issa, Hoor, Ayden, Liben, Borena, Kerreyu, Afar, , Bishari, Arabi, Rashaidi, Anafi, Kenani, Steppe, Sahraoui | Ethiopia, Sudan, Algeria | Osman et al., 2015; Legesse et al., 2018, Meghelli et al., 2020 |
|  | F: 102.3-111.0 | Jigjiga, Issa, Hoor, Ayden, Liben, Borena, Kerreyu, Afar, Aftout, Bishari, Arabi, Rashaidi, Anafi, Kenani, Steppe, Sahraoui | Ethiopia, Mauritania, Sudan, Algeria | Osman et al., 2015; Legesse et al., 2018; Ould Ahmed et al., 2022, Meghelli et al., 2020 |
|  | Combined: 109.1-113.0 | Steppe, Sahraoui, Bishari, Arabi, Rashaidi, Anafi, Kenani | Algeria, Sudan | Osman et al., 2015; Meghelli et al., 2020 |
| Neck girth | M: 61.3 | Steppe, Sahraoui | Algeria | Meghelli et al., 2020 |
|  | F: 60.6-70.6 | Steppe, Sahraoui, Aftout | Algeria, Mauritania | Meghelli et al., 2020; Ould Ahmed et al., 2022 |
|  | Combined: 61. | Steppe, Sahraoui | Algeria | Meghelli et al., 2020 |
| Ear length | M: 12.5-13.4 | Jigjiga, Issa, Hoor, Ayden, Liben, Borena, Kerreyu, Afar, Shinille, Nigerian Indigenous | Ethiopia, Nigeria | Yosef et al., 2014; Tandoh et al., 2018; Legesse et al., 2018 |
|  | F: 11.8-13.4 | Jigjiga, Issa, Hoor, Ayden, Liben, Borena, Kerreyu, Afar, Nigerian Indigenous | Ethiopia, Nigeria | Yosef et al., 2014; Tandoh et al., 2018; Legesse et al., 2018; |
|  | Combined: 10.4-13.36 | Jigjiga, Issa, Hoor, Ayden, Liben, Borena, Kerreyu, Afar, Shinille | Ethiopia | Yosef et al., 2014; Legesse et al., 2018; Kebede et al., 2022 |
| Face length | M: 50.6-59.7 | Jigjiga, Issa, Hoor, Ayden, Liben, Borena, Kerreyu, Afar, Bishari, Arabi, Rashaidi, Anafi, Kenani, Nigerian Indigenous | Sudan, Ethiopia, Nigerian | Osman et al., 2015; Legesse et al., 2018, Tandoh et al., 2018; |
|  | F: 50.6-58.1 | Aftout, Jigjiga, Issa, Hoor, Ayden, Liben, Borena, Kerreyu, Afar, Bishari, Arabi, Rashaidi, Anafi, Kenani, Nigerian Indigenous | Mauritania, Sudan, Ethiopia, Nigerian | Osman et al., 2015; Legesse et al., 2018; Tandoh et al., 2018; Ould Ahmed et al., 2022 |
|  | Combined: 48.2-58.8 | Steppe, Sahraoui, Bishari, Arabi, Rashaidi, Anafi, Kenani | Algeria, Sudan | Osman et al., 2015; Meghelli et al., 2020 |
| Head width | F: 28.0 | Aftout | Mauritania | Ould Ahmed et al., 2022 |
| Distance between eyes | M: 23.4-30.3 | Nigerian Indigenous, Shinille, Jigjiga, Issa, Hoor, Ayden, Liben, Borena, Kerreyu, Afar, Steppe, Sahraoui | Nigeria, Ethiopia, Algeria | Yosef et al., 2014; Tandoh et al., 2018; Meghelli et al., 2020 |
|  | F: 24.0-29.1 | Nigerian Indigenous, Shinille, Jigjiga, Issa, Hoor, Ayden, Liben, Borena, Kerreyu, Afar, Steppe, Sahraoui | Nigeria, Ethiopia, Algeria | Yosef et al., 2014; Tandoh et al., 2018; Legesse et al., 2018; Meghelli et al., 2020 |
|  | Combined: 23.7-27.2 | Shinille, Jigjiga, Issa, Hoor, Ayden, Liben, Borena, Kerreyu, Afar, Steppe, Sahraoui | Ethiopia, Algeria | Yosef et al., 2014; Legesse et al., 2018; Meghelli et al., 2020 |
| Hump length | M: 32.6-46.4 | Nigerian Indigenous, Jigjiga, Issa, Hoor, Ayden, Liben, Borena, Kerreyu, Afar | Nigeria, Ethiopia | Yosef et al., 2014; Tandoh et al., 2018; Legesse et al., 2018 |
|  | F: 32.5-45.5 | Nigerian Indigenous, Jigjiga, Issa, Hoor, Ayden, Liben, Borena, Kerreyu, Afar | Nigeria, Ethiopia | Yosef et al., 2014; Tandoh et al., 2018; Legesse et al., 2018 |
|  | Combined: 32.5-35.8 | Jigjiga, Issa, Hoor, Ayden, Liben, Borena, Kerreyu, Afar | Ethiopia | Yosef et al., 2014, Legesse et al., 2018 |
| Hump circumference | M: 112.9-153.1 | Nigerian Indigenous, Jigjiga, Issa, Hoor, Ayden, Liben, Borena, Kerreyu, Afar | Nigeria, Ethiopia | Yosef et al., 2014; Tandoh et al., 2018; Legesse et al., 2018 |
|  | F: 78.5-142.3 | Nigerian Indigenous, Jigjiga, Issa, Hoor, Ayden, Liben, Borena, Kerreyu, Afar | Nigeria, Ethiopia | Yosef et al., 2014; Tandoh et al., 2018; Gebremariam et al., 2020 |
|  | Combined: 136.4-142.3 | Jigjiga, Issa, Hoor, Ayden, Liben, Borena, Kerreyu, Afar | Ethiopia | Yosef et al., 2014; Legesse et al., 2018 |
| Hip width | M: 42.5-44.6 | Gelleb, Jigjiga, Issa, Hoor, Ayden, Liben, Borena, Kerreyu, Afar | Ethiopia | Yosef et al., 2014; Legesse et al., 2018 |
|  | F: 42.5-47.7 | Gelleb, Jigjiga, Issa, Hoor, Ayden, Liben, Borena, Kerreyu, Afar | Ethiopia | Yosef et al., 2014; Legesse et al., 2018 |
|  | Combined: 42.5-47.1 | Gelleb, Jigjiga, Issa, Hoor, Ayden, Liben, Borena, Kerreyu, Afar | Ethiopia | Yosef et al., 2014; Legesse et al., 2018 |
| Tail Length | M: 51.7-70.2 | Steppe, Sahraoui, Nigerian Indigenous, Bishari, Arabi, Rashaidi, Anafi, Kenani, Gelleb, Jigjiga, Issa, Hoor, Ayden, Liben, Borena, Kerreyu, Afar | Algeria, Nigeria, Sudan, Ethiopia | Yosef et al., 2014; Osman et al., 2015; Tandoh et al., 2018; Legesse et al., 2018; Meghelli et al., 2020 |
|  | F: 55.1-67.8 | Steppe, Sahraoui, Nigerian Indigenous, Bishari, Arabi, Rashaidi, Anafi, Kenani, Gelleb, Jigjiga, Issa, Hoor, Ayden, Liben, Borena, Kerreyu, Afar | Algeria, Nigeria, Sudan, Ethiopia | Yosef et al., 2014; Osman et al., 2015; Tandoh et al., 2018; Legesse et al., 2018; Meghelli et al., 2020 |
|  | Combined: 53.4-68.6 | Steppe, Sahraoui, Bishari, Arabi, Rashaidi, Anafi, Kenani, Gelleb, Jigjiga, Issa, Hoor, Ayden, Liben, Borena, Kerreyu, Afar | Algeria, Sudan, Ethiopia | Yosef et al., 2014; Osman et al., 2015; Legesse et al., 2018; Meghelli et al., 2020 |

M= male; F= female
