# Supplementary material for: Exploiting morphobiometric and genomic variability of African indigenous camel populations-A review
Source: Front Genet. 2022 Dec 12;13:1021685. doi: 10.3389/fgene.2022.1021685 (PMC9791103; doi:10.3389/fgene.2022.1021685)
Supplement: Supplementary file 2 [file DataSheet1.PDF]

COAT COLOURS OF AFRICAN CAMELS

Dark-Brown

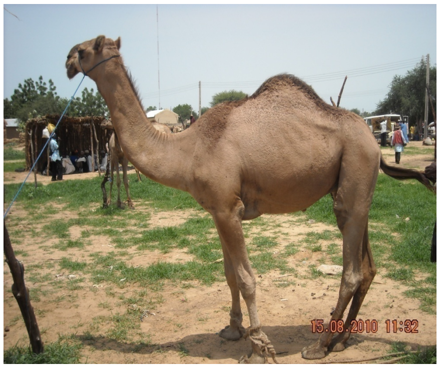

Sand-Brown

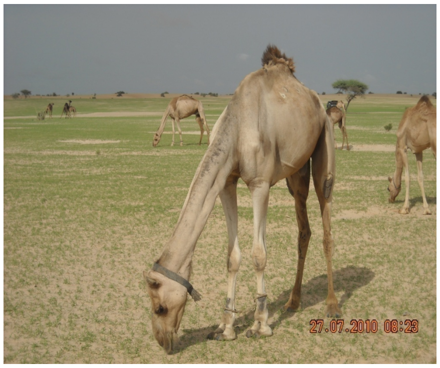

Source: Abdussamad et al. (2015)

Grey-White

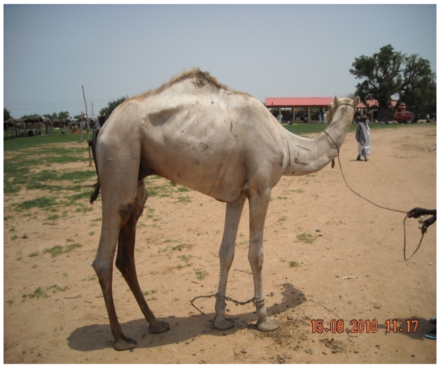

White

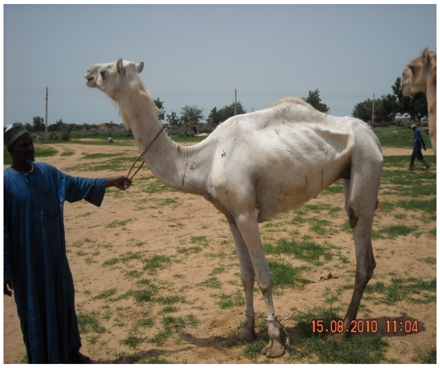

Pied Coloured

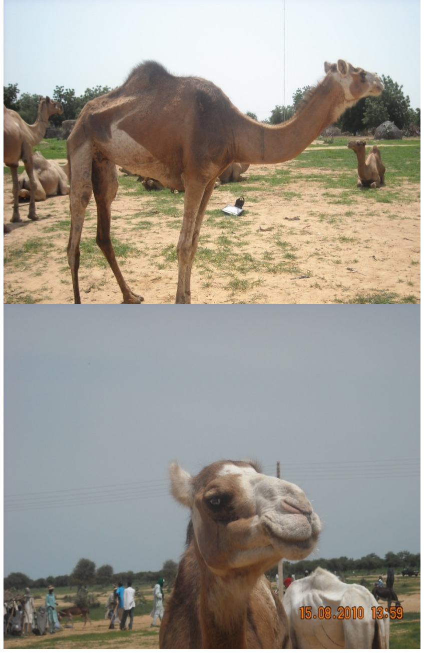

Brown-Black

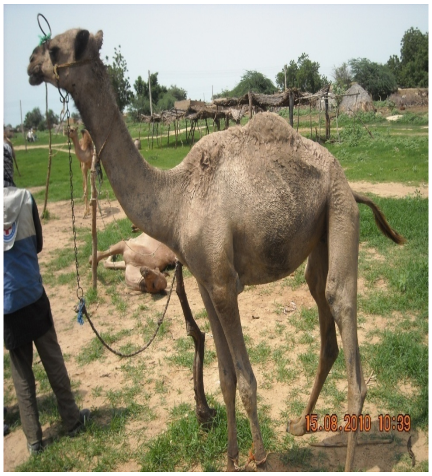

Source: Abdussamad et al. (2015)

Black

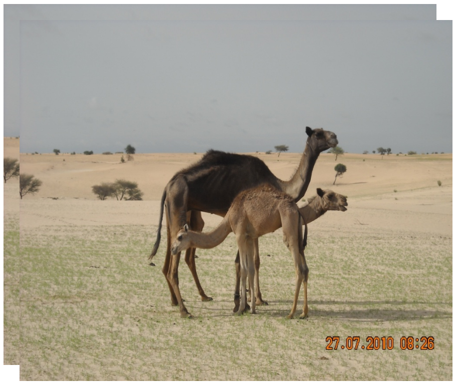

Source: Abdussamad et al. (2015)

Brown

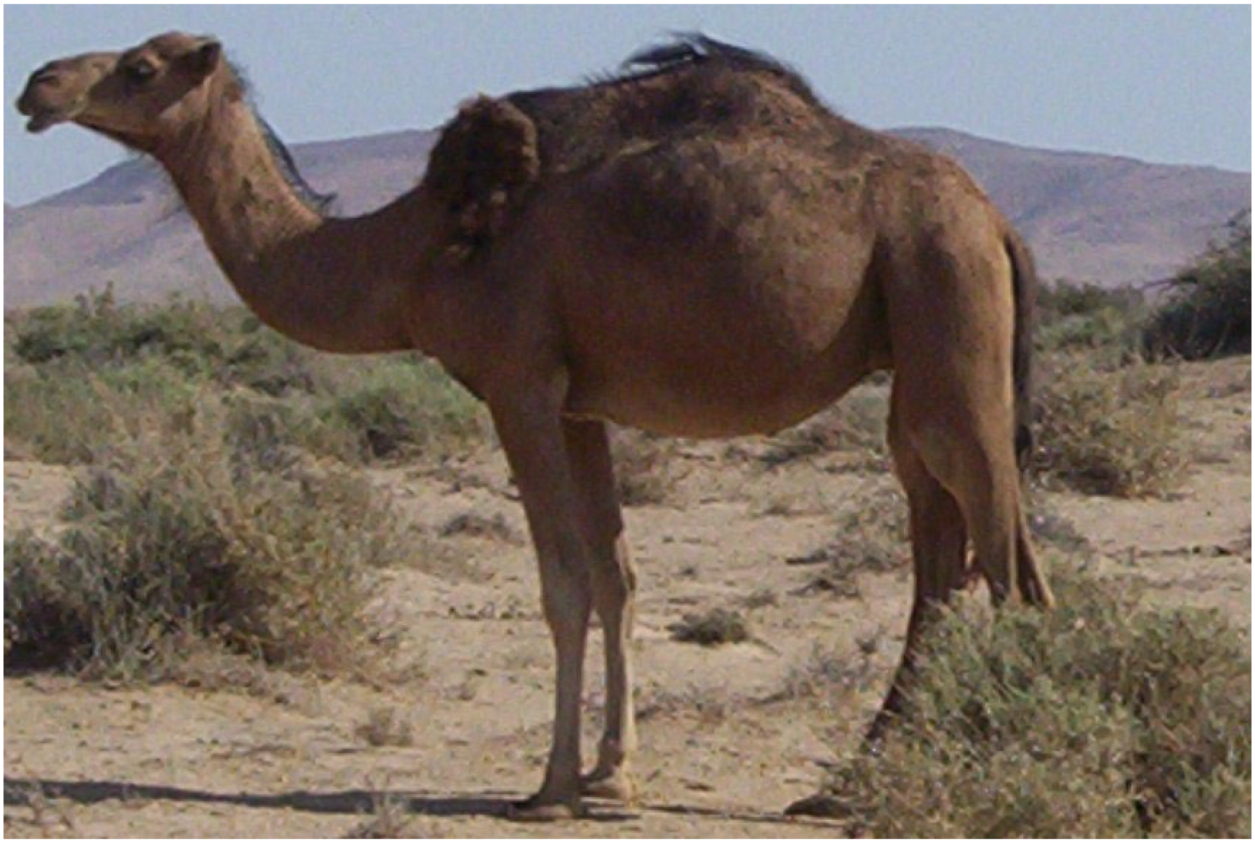

Source: Chniter et al. (2013)

Fawn

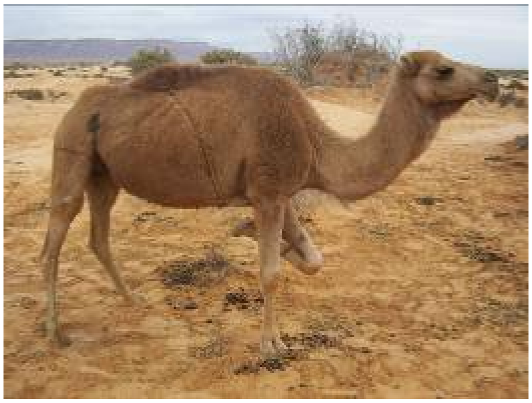

Source: Chniter et al. (2013)

Yellow

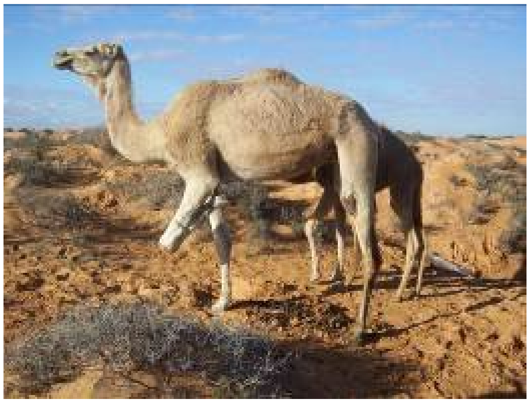

Source: Chniter et al. (2013)

Creamish

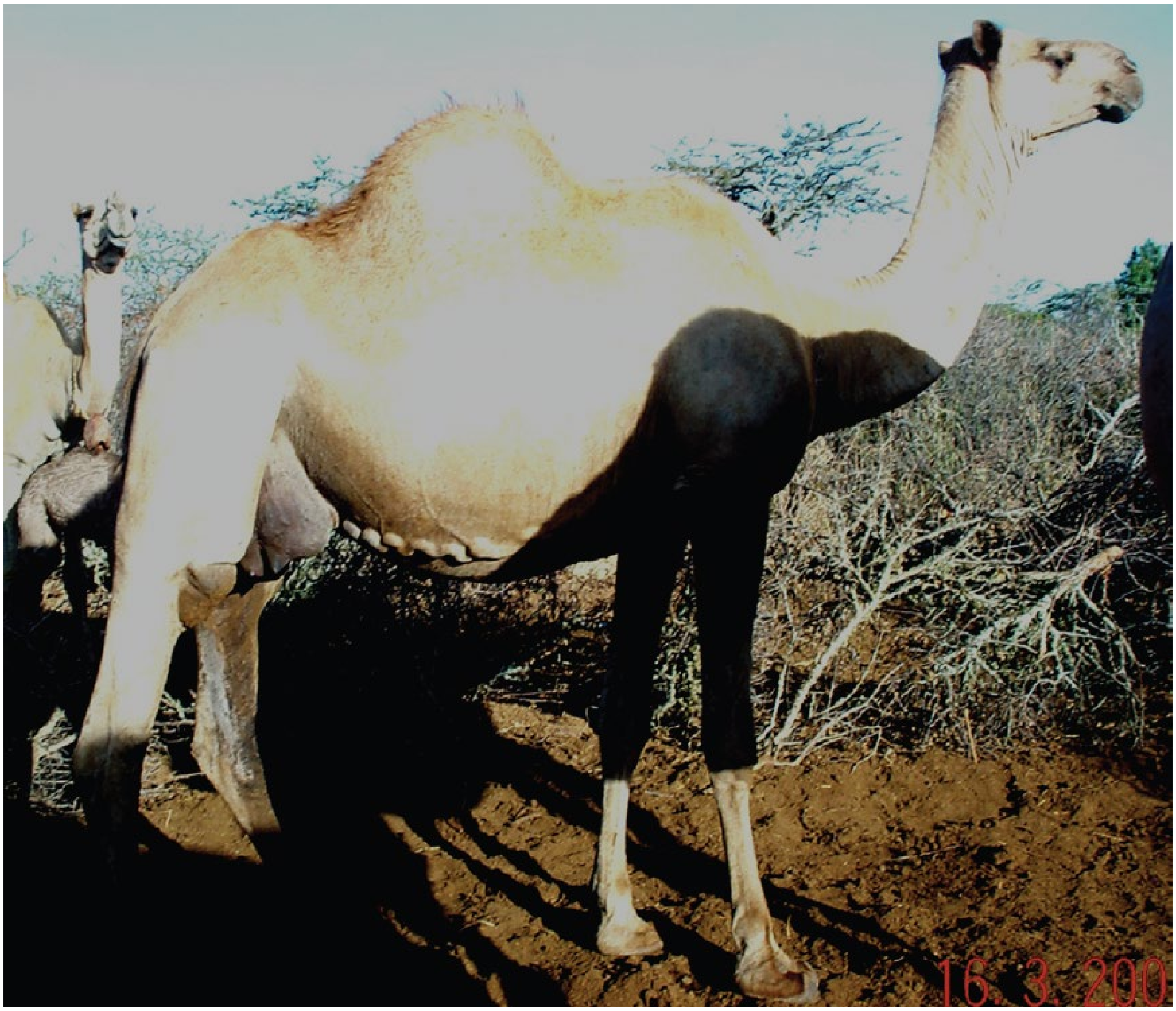

Source: Tura et al. (2008)

Red

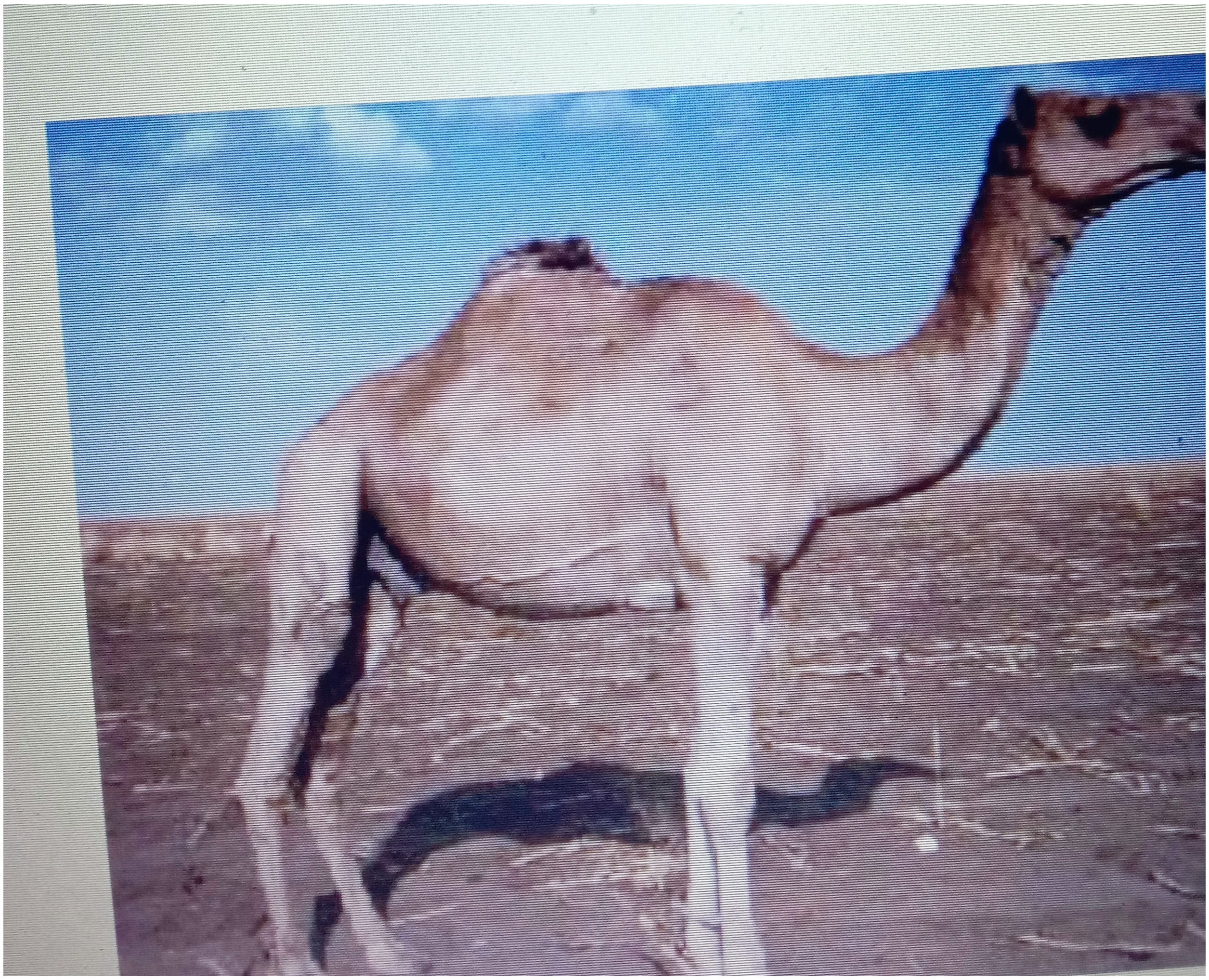

Source: Mohamed (2010).

HAIR TYPES OF AFRICAN CAMELS

Rough hair

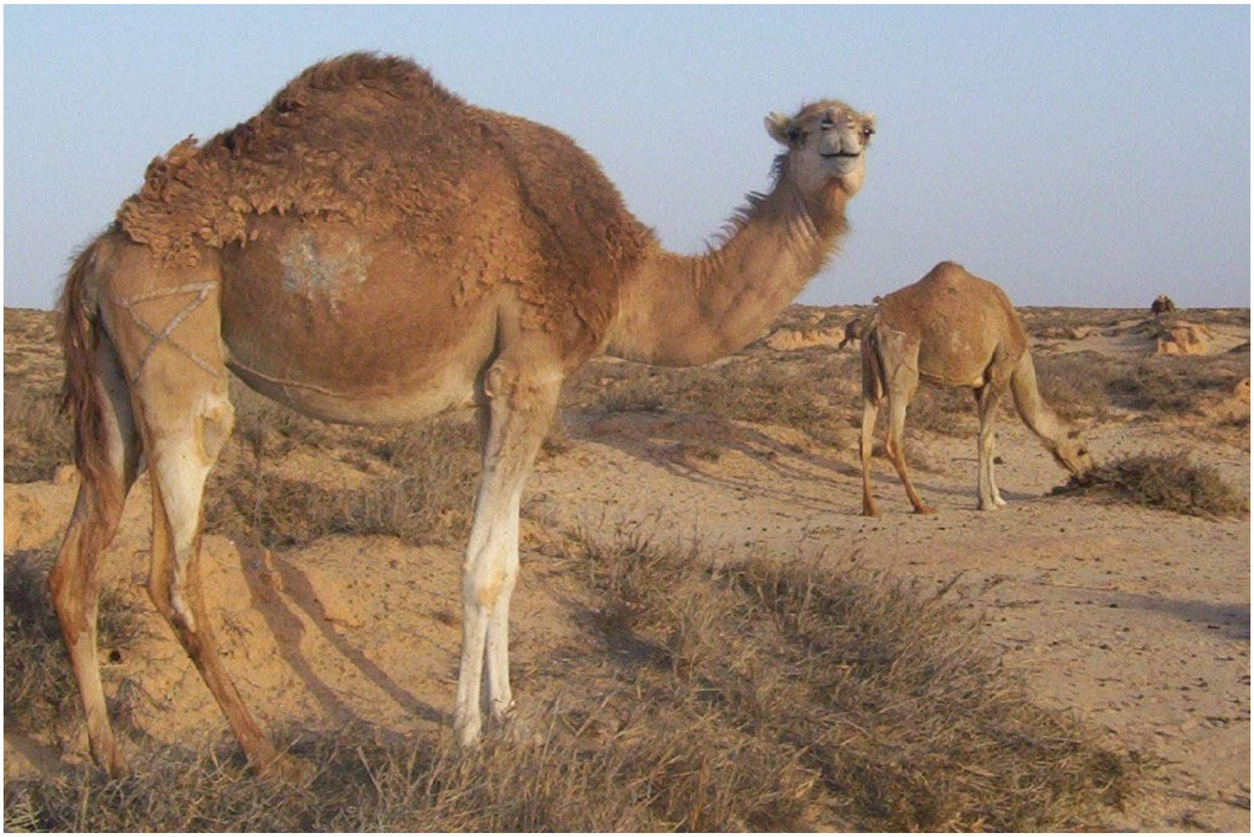

Source: Chniter et al. (2013)

Smooth

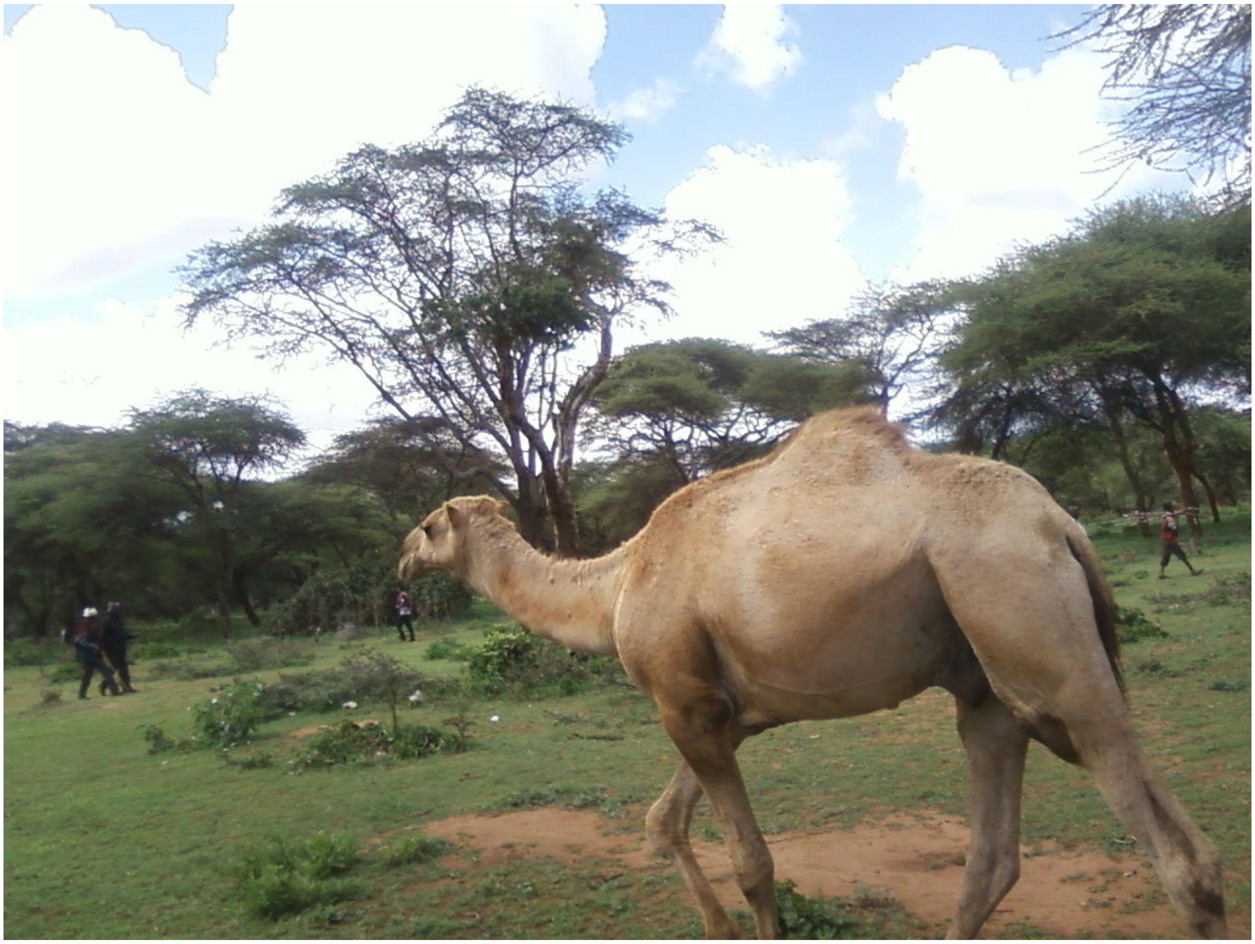

Source: Bekele et al. (2018).
